# mSphere of Influence: The power of polymicrobial partnerships in chronic infection research

**DOI:** 10.1128/msphere.00434-24

**Published:** 2024-08-20

**Authors:** Chelsey M. VanDrisse

**Affiliations:** 1 Department of Genetics, University of Georgia, Athens, Georgia, USA; University of Georgia, Athens, Georgia, USA

**Keywords:** *Pseudomonas*, biofilms, polymicrobial, cystic fibrosis

## Abstract

Chelsey VanDrisse works in the field of microbial physiology, studying how acylation of small molecules and proteins affects the development of *Pseudomonas* biofilms. In this mSphere of Influence article, she reflects on the paper “Community composition shapes microbial-specific phenotypes in a cystic fibrosis polymicrobial model system” by Jean-Pierre et al. This paper prompted her to reassess her approach to studying antibiotic tolerance and her design of experiments that search for disease-relevant mutants and phenotypes in the laboratory.

## COMMENTARY

The difficulty of eradicating pathogens in chronic infections is in part due to the intricate and variable host environment that starkly differs from the controlled conditions we study in the laboratory. Nonetheless, researchers have successfully attempted to close this gap by developing culture media that mimic host nutrient availability (1, 2) by growing cells in the presence of immune cells such as macrophages (3, 4) and by using animal models (5, 6). In all such systems, however, we often focus on the physiology and metabolism of single species, yet bacteria rarely exist in isolation in the natural world (7). Developing new polymicrobial model systems that mimic real-world community compositions represents a radical shift away from how controlled laboratory experiments have traditionally been conducted, a shift that researchers hope can provide fresh insights into why some chronic infections are so difficult to eradicate. One devastating example of a recurrent, chronic bacterial infection is found in the lungs of cystic fibrosis (CF) patients. In patients with CF, a mutation in the human CFTR gene results in a thickening of mucus within the lungs (8). This nutrient-rich mucus environment provides a niche for bacteria to thrive, ultimately leading to pulmonary decline. Often, a single species dominates a bacterial community in the lungs of CF patients (9), driving many past and current research efforts to focus on understanding and undermining that dominant species. However, this of course assumes that the presence of the minor species would have a commensurate effect on the experiment outcome, an assumption that was tested by Jean-Pierre et al. in their work titled “Community composition shapes microbial-specific phenotypes in a cystic fibrosis polymicrobial model system (10).” By leveraging clinical sample data from CF patients, they aimed to create clinically relevant *in vitro* models to study the antibiotic sensitivity of microbial communities and compare their results to conclusions made from identical pure-culture experiments.

Jean-Pierre et al. developed a sophisticated polymicrobial *in vitro* model system incorporating four microbes frequently coexisting in CF patients: *Pseudomonas*, *Staphylococcus*, *Streptococcus*, and *Prevotella*. Their objective was to investigate whether unknown factors within these mixed microbial communities enable bacteria to survive exposure to a clinically relevant CF antibiotic, tobramycin. Their initial findings falsified the long-standing and implicit assumptions of pure-culture experiments: the four-species community exhibited markedly different survival outcomes after tobramycin treatment compared to each species cultured individually but not always in the way you might expect. For instance, when *Pseudomonas* was grown alone in a biofilm, it exhibited a multi-log reduction in viability upon tobramycin treatment. Within the four-species community, *Pseudomonas* was completely eradicated, undetectable post-treatment. On the other hand, *Streptococcus* displayed the opposite behavior, being eliminated below detectable levels when grown alone in the presence of tobramycin yet surviving within the polymicrobial community, showing no significant difference between treated and untreated conditions. Additionally, *Prevotella*, which failed to grow in isolation under CF-mimicking conditions, only grew successfully when part of the mixed-species community. Jean-Pierre et al. also investigated the antibiotic survival of *Pseudomonas* mutants exposed to tobramycin. They found that deletion of certain genes changed the survival of *Pseudomonas* compared to wild type in the mixed community. Besides raising many new and exciting questions, these findings compellingly illustrate that studying bacteria in monoculture, while informative about individual species’ physiology, fails to capture the complex interactions and behaviors that emerge in polymicrobial environments. Jean-Pierre et al. suggest a new standard of considering microbial community dynamics to fully understand and effectively treat chronic infections, and have provided the field with one that can be further investigated by others.

Jean-Pierre et al.’s work opened my eyes to novel ways of studying *Pseudomonas* within my own research. My tried-and-true strategy of classic physiological and genetic studies is an algorithmic approach of knocking out a gene and searching for phenotypes in well-controlled contexts. Despite supplying a wealth of basic knowledge over the last century, it is now well appreciated that this style of reductionism may generate knowledge that is not contextually relevant in many real-world settings (11). Where do we go from here? The answer does not appear to be a radical new approach to science but instead innovative new methods that apply the relevant context within experiments. In this spirit, we must seek to understand the metabolic and physical dynamics that bacteria experience in their natural environments, as these provide vital clues to what we might be overlooking in the laboratory. In my future work, if I am stuck trying to find a phenotype in *Pseudomonas*, I will remember the work of Jean-Pierre et al. and ask not what I need to do to *Pseudomonas* but, rather, who should I include? In shifting our focus from isolated species to their interactions within complex communities, we will likely unlock new secrets to effectively combat chronic infections and antibiotic tolerance.
